# Practical Recommendations for Indians on Sunscreen Use—A Modified Delphi Consensus by Indian Sunscreen Forum (PRISM‐ISF)

**DOI:** 10.1111/jocd.70441

**Published:** 2025-09-16

**Authors:** Malavika Kohli, C. R. Srinivas, Abir Saraswat, Anil Ganjoo, Atul Kathed, Geeta Patel, Indrashis Podder, D. S. Krupashankar, Maleeka Sachdev, Maya Vedamurthy, Rajat Kandhari, Rajetha Damisetty, Renita Rajan, Sanjeev Aurangabadkar, Dhiraj Dhoot, Ashwin Balasubramanian, Saiprasad Patil, Hanmant Barkate

**Affiliations:** ^1^ Breach Candy Hospital, and Jaslok Hospital Mumbai Maharashtra India; ^2^ Department of Dermatology Kalinga Institute of Medical Sciences Bhubaneswar Odisha India; ^3^ Department of Dermatology KMC Manipal Manipal Karnataka India; ^4^ Indushree Skin Clinic Lucknow Uttar Pradesh India; ^5^ Skinnovation Clinics New Delhi India; ^6^ Drkathed's Clinic, Care CHL Hospital Indore India; ^7^ Zahra Skin and Laser Clinic Ahmedabad Gujarat India; ^8^ Department of Dermatology College of Medicine and Sagore Dutta Hospital Kolkata West Bengal India; ^9^ Mallige Hospital Bangalore India; ^10^ Sachdev Clinics Chandigarh India; ^11^ Department of Dermatology RSV Skin and Research Centre Chennai Tamil Nadu India; ^12^ Dr Kandhari's Skin and Dental Clinic, Veya Aesthetics New Delhi India; ^13^ Dr. Rajetha's Mohana Skin & Hair Clinic Hyderabad Telangana India; ^14^ Render Skin & Hair Chennai India; ^15^ Aurangabadkar's Skin & Laser Clinics Hyderabad Telangana India; ^16^ Department of Global Medical Affairs Glenmark Pharmaceuticals Limited Mumbai Maharashtra India

**Keywords:** chemical, consensus, disease, India, mineral, skin type, sunscreen

## Abstract

**Background:**

Ultraviolet (UV) radiation is a major contributor to photoaging, pigmentary disorders, and photocarcinogenesis. While sunscreens remain central to photoprotection, clinicians in India find it challenging to choose a sunscreen due to the wide variability in skin types, dermatologic conditions, climates, and formulation preferences. Selecting a sunscreen that balances efficacy, cosmetic acceptability, affordability, and patient adherence remains a practical dilemma.

**Objective:**

To develop consensus‐based, practical recommendations for sunscreen selection and use in Indian dermatologic practice, addressing gaps in clinical guidance and contextual challenges.

**Methods:**

A panel of 14 dermatologists from across India, forming the Indian Sunscreen Forum (ISF), was convened through an in‐person scientific meeting followed by iterative virtual discussions using a modified Delphi technique. The panel reviewed relevant scientific literature, real‐world clinical practices, and patient‐centric factors affecting sunscreen use in India.

**Results:**

Consensus was achieved on preferred sunscreen formulation types (physical, chemical, and hybrid), application techniques, frequency, quantity, and specific recommendations across dermatologic indications including melasma, photodermatoses, acne, and post‐procedure care. The panel also addressed considerations for special populations such as children, pregnant women, and individuals with sensitive skin. Safety concerns around systemic absorption and environmental impact were also discussed.

**Conclusion:**

This consensus statement provides Indian clinicians with evidence‐informed, context‐specific guidance for recommending sunscreens, helping them streamline decision‐making, improve patient compliance, and align photoprotection strategies with dermatologic and public health priorities in India.

## 1 Introduction

Sunlight exposure is widely recognized for its mood‐enhancing properties and health benefits, including beneficial effects in conditions such as rickets, psoriasis, eczema, and vitiligo, as well as promoting vitamin D synthesis. However, over time, the adverse effects of excessive sun exposure have become increasingly evident. The skin‐acting as the body's primary interface with the environment is constantly exposed to various oxidative stressors, including solar radiation and environmental pollutants such as vehicle emissions, cigarette smoke, halogenated hydrocarbons, heavy metals, and ozone. As the body's first line of defense, it plays a crucial role in protecting against these external threats [1, 2].

The solar spectrum includes ultraviolet (UV) radiation within the 100–400 nm wavelength range. However, only UVA (315–400 nm) and UVB (280–315 nm) reach the Earth's surface, as UVC (100–280 nm) is entirely absorbed by the ozone layer and does not pose a clinical risk [3, 4]. While acute exposure to UV radiation can lead to erythema, sunburn, and photo‐immunosuppression, chronic exposure contributes to photoaging and increases the risk of skin cancer [3, 5].

Implementing sun protection strategies—such as seeking shade, wearing protective clothing, hats, sunglasses, and applying a sunscreen—is essential in minimizing UV‐related skin damage. Among these measures, sunscreens play a pivotal role in shielding the skin from harmful ultraviolet radiation [4, 5].

An effective approach to mitigating the harmful effects of sun exposure involves using broad‐spectrum sunscreens that provide protection against both UVA and UVB radiation [3, 4, 5, 6]. The primary objective of sunscreen formulations is to safeguard the skin while ensuring high safety standards, optimal skin compatibility, and a pleasant user experience [4].

Dermatologists play a vital role in educating patients about photoprotection, including the appropriate selection and application of sunscreens. By raising awareness and promoting proper sun protection habits, the risks of both acute and long‐term UV‐induced skin damage can be significantly reduced. But there are various factors influencing the choice of sunscreen, like disease profile, skin types and phototypes, climatic conditions, lifestyle factors, cosmetic elegance, affordability, among many others. Given the complexities involved in selecting an appropriate sunscreen, this consensus statement aims to highlight the importance of topical photoprotection, the challenges associated with sunscreen formulations, and the necessity of stringent regulatory measures to ensure product efficacy, especially in Indian patients. Table 1 contains some commonly mentioned parameters on the sunscreen label along with their definitions.

**TABLE 1:** Commonly mentioned parameters on the sunscreen label.

Term	Definition
Sun protection factor (SPF) [7]	It is the numerical ratio between the minimal erythemal dose (MED) of sunscreen‐protected skin, applied in the amount of 2 mg/cm^2^ and the Minimal Erythemal dose of unprotected skin, a mathematical relation that can be represented by the following equation: SPF = MED (protected skin)/MED (unprotected skin)
Protection grade of UVA (PA) [8]	The PA system is based on the Persistent Pigment Darkening (PPD) method, which measures how much UVA‐induced tanning is prevented. It is given a “+” score as follows: 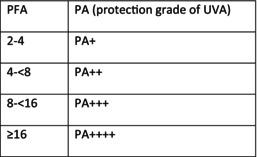
Boots star [8]	It is an in vitro measurement of the ratio of UVA (320–400 nm) absorbance over UVB (290–320 nm) absorbance. *** (3 stars): UVA to UVB ratio between 0.6 and 0.79. **** (4 stars): UVA to UVB ratio between 0.8 and 0.89. ***** (5 stars): UVA to UVB ratio between 0.9 or higher.
Critical wavelength [8]	The wavelength below which 90% of the sunscreen's UV absorbency occurs
Broad spectrum sunscreen [9]	Critical wavelength > 370 nm AND UVA protection factor > 4
Water‐resistant sunscreen [9]	Maintains the label SPF value after two sequential immersions in water for 20 min (40 min)
Very water‐resistant sunscreen [9]	Maintains the label SPF value after four sequential immersions in water for 20 min (80 min)

**FIGURE 1:**
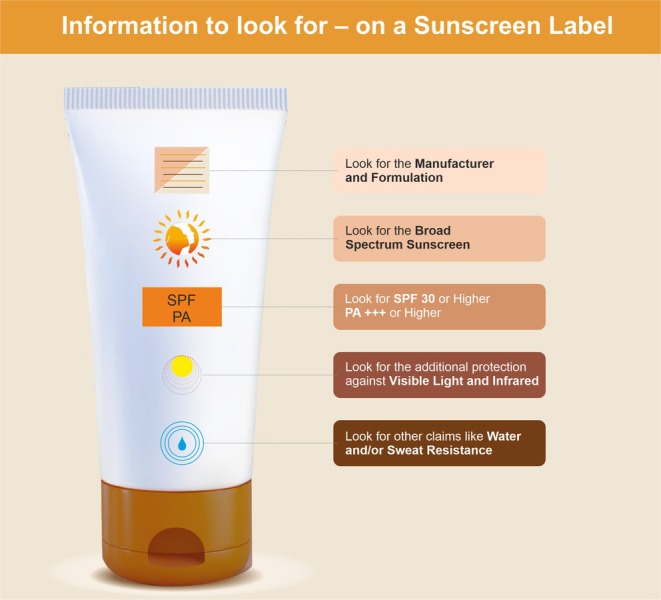
Information to look for on a sunscreen label.

Figure 1 shows the labeling of a sunscreen detailing the formulation, UVB, UVA, water resistance, and identification as a broad‐spectrum sunscreen.

### 1.1 Definitions of Common Dermatological Disorders Where a Sunscreen Is Prescribed

Photosensitivity refers to various symptoms, diseases, and photo dermatoses caused or exacerbated by exposure to sunlight. Photosensitive conditions are classified into five categories, which include [10]:

- Primary or autoimmune photodermatosis
- Exogenous or drug/chemical‐induced photodermatosis
- Photo‐exacerbated or photo‐aggravated dermatoses
- Metabolic photodermatosis
- Genetic photodermatosis

## 2 Materials and Methods

### 2.1 Consensus Development Process

To establish an expert‐driven guideline on sunscreen use, the Indian Sunscreen Forum (ISF) was formed, comprising 14 leading dermatologists, each with over 20 years of clinical experience across India. These experts were selected based on their clinical expertise and their commitment to advancing public awareness regarding the importance of sun protection. The ISF's primary mission was to not only educate the Indian population about sunscreen use but also to develop practical, evidence‐based consensus statements for clinical implementation.

On May 26, 2024, all forum members convened for an in‐person meeting to formally establish the ISF. During the session, panelists presented scientific findings and shared their clinical experiences to provide an up‐to‐date perspective on the benefits and potential risks of sunscreen application. Through detailed discussions, the group reached an agreement on the necessity of developing consensus statements covering key aspects such as the role of sunscreens in dermatological diseases, the impact of UV radiation, the influence of visible light, blue light, and infrared radiation on the skin, various sunscreen formulations and their effectiveness, sunscreen use in specific populations, and the overall effects of sunscreen application on human health.

Following the meeting, an extensive literature review was conducted using a combination of the following Medical Subject Headings (MeSH) terms and free‐text words including: “ultraviolet radiation,” “ultraviolet spectrum,” “sunscreen guidelines,” “sunscreen consensus,” “Sunscreen,” “Sun protection,” “photoprotection,” “sunburn,” “broad‐spectrum sunscreen,” “sunscreen application,” “water‐resistant sunscreen,” “iron oxide,” “dermatologic procedures,” “noncomedogenic,” “acne vulgaris,” “melasma,” “postinflammatory hyperpigmentation,” “visible light protection,” “infrared light protection,” “photodermatoses,” “photosensitive disorders,” “chemical sunscreen,” “mineral sunscreen,” “vitamin D deficiency,” “pregnancy,” “nanoparticle,” “endocrine disruption,” “environment,” “coral reef,” and “antioxidant.”

There were initially 50 statements that were structured to be responded to by the expert panelists based on a 5‐point Likert Scale (5—Strongly Agree, 4—Agree, 3—Neutral, 2—Disagree, 1—Strongly Disagree). These statements underwent pre‐validation by a panel moderator. The consensus process utilized a modified Delphi technique, with two rounds of anonymous voting conducted on a virtual platform. The responses were collected anonymously. Experts consented to the aggregate analysis of their responses. Consensus was defined as ≥ 75% agreement on a 5‐point Likert scale. Statements were then categorized into positive recommendations, where ≥ 75% of participants agreed or strongly agreed, or negative recommendations, where ≥ 75% disagreed or strongly disagreed. Statements with 50%–74% agreement were considered to have near consensus, while those with < 50% agreement were classified as having no consensus. Statements not achieving 75% concordance were revisited for further discussion.

## 3 Results

For the purpose of sunscreen recommendations, experts decided to segregate various dermatological disorders into the following three categories of disorders, which will be referred to in the subsequent sections:

- Photosensitive disorders: Dermatoses that manifest on exposure to sunlight, like porphyrias, PMLE, SLE, DLE, drug‐induced sun sensitivity, and genetic photosensitivity disorders.
- Photoaggravated disorders: Dermatoses that are present even without sun exposure but worsen when exposed to sunlight like atopic dermatitis, acne vulgaris, pemphigus, and psoriasis.
- Photopigmentary disorders: Dermatoses like melasma and PIH.

After the vote on the initial 50 statements through the online platform, expert opinions were gathered using the modified Delphi method during two virtual meetings, ensuring a structured, collaborative approach toward finalizing the consensus statements.

**FIGURE 2:**
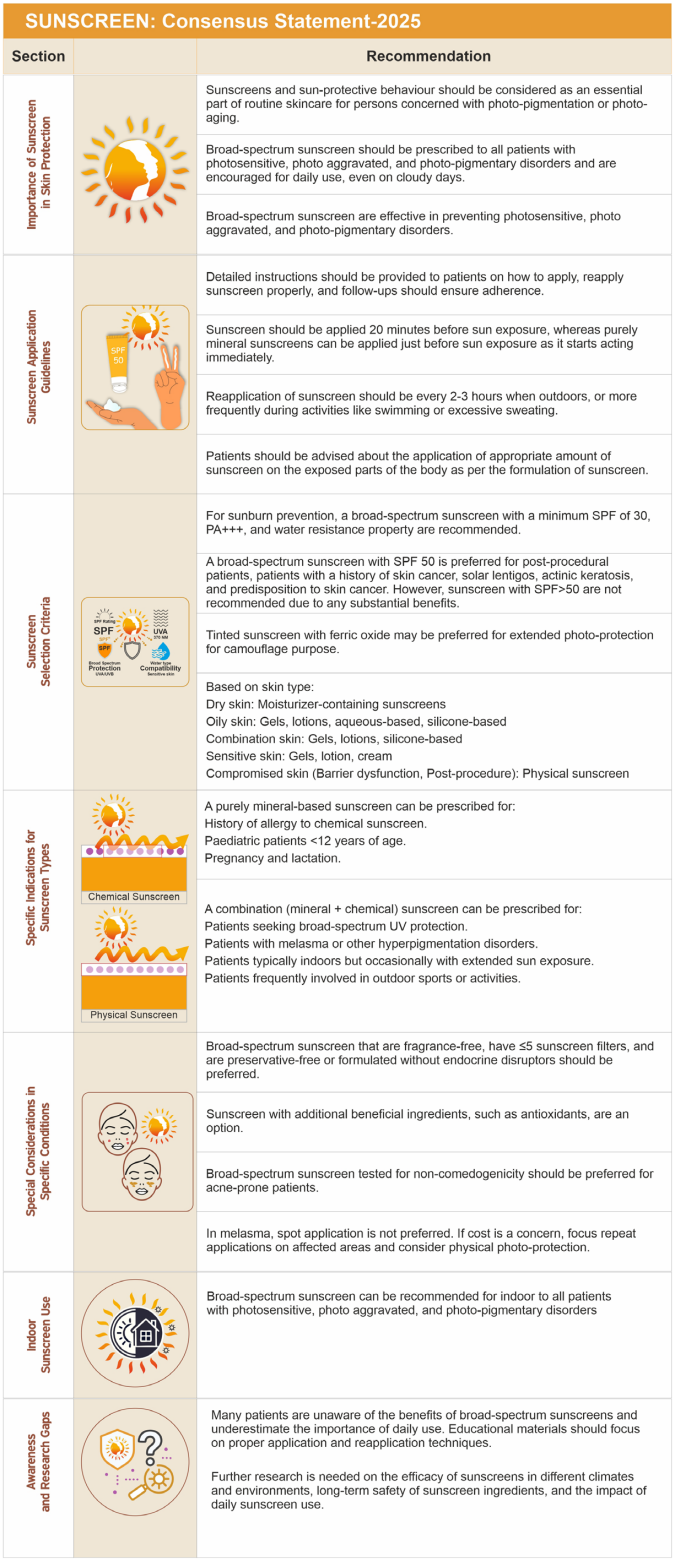
Sunscreen consensus statements.

After the first meeting, 14 statements reached consensus, 10 statements were excluded, 20 statements were to be modified, and 6 statements required further literature review. In the second meeting, the remaining statements that achieved consensus were clustered to finally form 18 consensus statements under seven sections: (i) importance of a sunscreen in skin protection, (ii) sunscreen application guidelines, (iii) sunscreen selection criteria, (iv) specific indications for sunscreen types, (v) special considerations in specific conditions, (vi) indoor sunscreen use, and (vii) awareness and research gaps, which are shared in Figure 2. The status of statements at various stages of the modified delphi consensus is shared as Appendix S1.

## 4 Discussion

The following section provides an in‐depth perspective based on the discussion by the expert panel on the consensus statements.

### 4.1 The Importance of a Sunscreen in Sun Protection

The solar electromagnetic spectrum that reaches the Earth's surface consists of three main types of radiation: UV, visible light (VL), and infrared (IR) [11]. UV radiation is further divided into UVC (100–280 nm), UVB (280–315 nm), and UVA (315–400 nm). While UVC is blocked by the stratospheric ozone layer, UVA and UVB penetrate the atmosphere and impact human skin. Approximately 95% of UV radiation exposure consists of UVA, which penetrates deeper into the skin, reaching the papillary dermis, while UVB, though less penetrating, causes more immediate damage at the epidermal level [12].

UV radiation causes both immediate and long‐term effects on the skin. Short‐term effects include sunburn (erythema), tanning, and immune suppression. Long‐term consequences involve photoaging, prolonged immunosuppression, and photocarcinogenesis [13, 14]. The initial reaction to UV radiation exposure is erythema, primarily due to UVB‐induced DNA damage. This triggers inflammatory pathways that produce mediators such as prostaglandins, cytokines, chemokines, histamine, and nitric oxide [15].

UVA plays a significant role in immediate pigment darkening by oxidizing and polymerizing melanin precursors. Both UVA and UVB contribute to delayed tanning [16]. While UVB exposure primarily leads to acute skin responses, UVA is largely responsible for chronic damage through oxidative stress, leading to collagen degradation and premature aging. Reactive oxygen species (ROS) generated by UVA exposure activate transcription factors like Activator Protein‐1 and Transforming Growth Factor‐β, further accelerating skin aging [17].

Unlike most carcinogens, UV radiation is classified as a “complete carcinogen” because it not only initiates mutations but also promotes tumor progression [18, 19]. Chronic exposure results in genetic damage through direct UVB absorption or indirect oxidative stress caused by UVA [13]. Additionally, UV radiation weakens immune surveillance, allowing cancerous cells to proliferate and metastasize [20].

Recent studies indicate that VL and IR radiation also contribute to free radical generation in the skin following sun exposure [5, 21]. IR radiation transmits heat, raising skin temperature and triggering the release of inflammatory mediators, which can independently stimulate melanogenesis. This interaction between IR, UV, and VL plays a significant role in harmful sunlight‐induced effects [3, 21, 22, 23].

Near‐infrared (IR‐A, 750–1400 nm) and VL can induce pigmentation even in the absence of UV exposure [3]. VL, particularly in darker skin tones, has a pronounced photobiological effect, contributing to hyperpigmentation and prolonged skin darkening [22, 24]. Research suggests that VL‐induced pigmentation lasts longer and is more intense than UVA1‐induced pigmentation. Furthermore, combined exposure to VL and UVA1 can lead to erythema in fair‐skinned individuals, a reaction previously associated primarily with UVB and UVA2 [23, 24].

A study also found that shorter wavelengths of VL directly stimulate melanocytes, with opsin‐3 acting as a blue light sensor, providing a potential target for preventing visible light‐induced hyperpigmentation [22, 23]. Notably, visible light‐induced pigmentation is more persistent in individuals with darker skin tones (phototype > III) [23].

**FIGURE 3:**
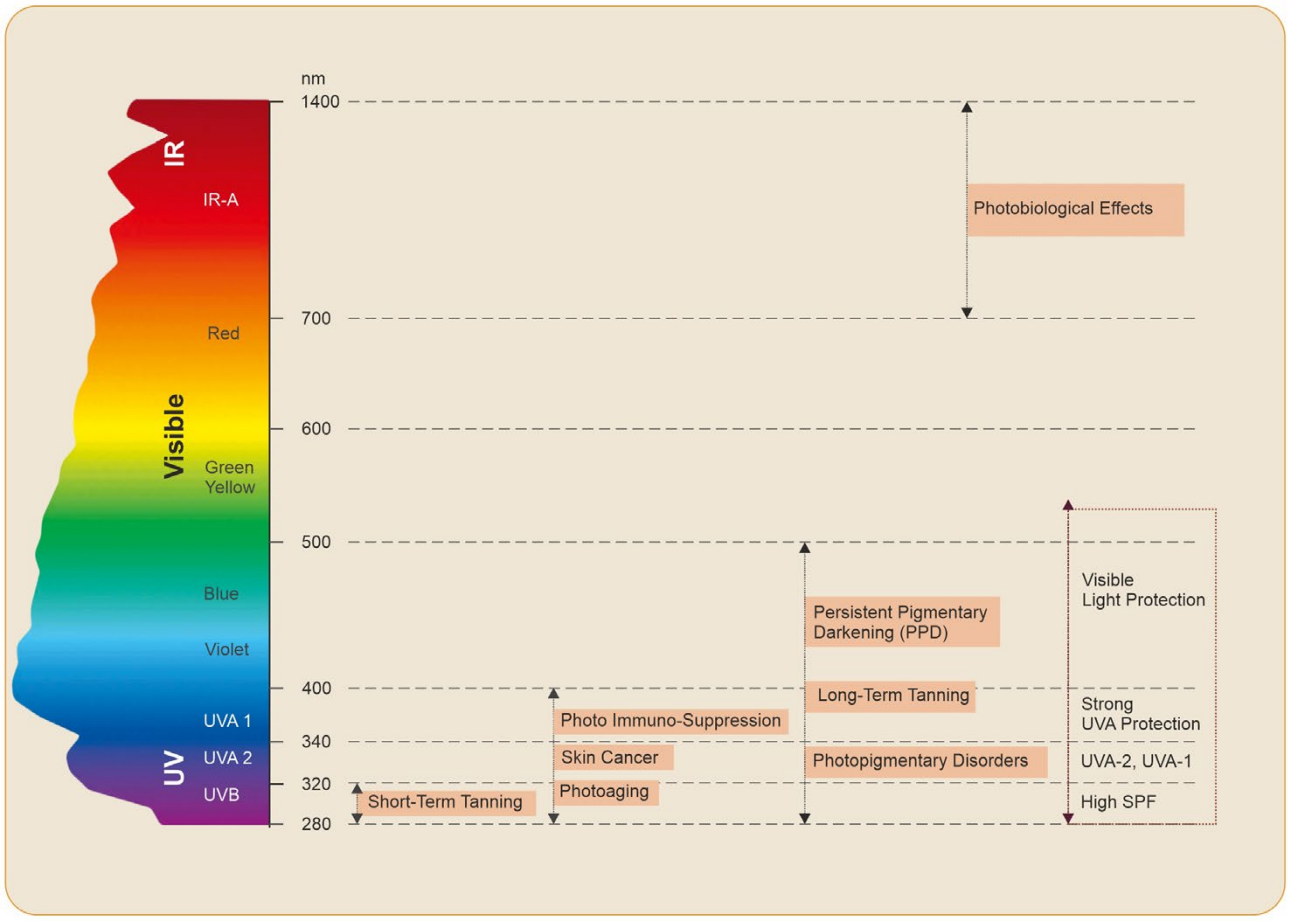
The impact of the electromagnetic spectrum on dermatological conditions. The Y‐axis denotes the wavelength of the type of radiation [1].

The relationship between different wavelengths of solar radiation and their role in photocarcinogenesis [25], photo‐immunosuppression [3, 26], photoaging [5, 24], and photodermatosis exacerbation [5, 26] is well documented in Figure 3.

#### 4.1.1 Practical Recommendations

1. UV radiation: The panel unanimously agreed that UVB is primarily responsible for sunburn and photocarcinogenesis, while UVA is a key factor in skin aging.
2. Visible light protection: The members recognized that visible light contributes to skin damage, including hyperpigmentation. Given that many electronic devices, such as laptop screens, emit blue light, they recommended protection through tinted sunscreens or broad‐spectrum sunscreens.
3. Infrared radiation protection: The panel acknowledged that IR radiation is linked to dermal inflammation, aging, and carcinogenesis. Individuals frequently exposed to IR, such as those who work in high‐heat environments like kitchens, require additional protection. In such cases, broad‐spectrum sunscreens or sunscreens with antioxidant properties are advisable.

In conclusion, comprehensive sun protection should account not only for UV radiation but also for visible light and infrared radiation. Using a broad‐spectrum sunscreen or formulations with additional protective agents can help mitigate the harmful effects of prolonged sun exposure, not only in normal individuals but also in patients associated with skin disorders.

### 4.2 Sunscreen Application Guidelines

Sunscreen formulation selection should be individualized based on skin type to ensure optimal efficacy and safety.

1. For oily or acne‐prone skin, noncomedogenic, oil‐free formulations such as gels, aqueous lotions, or silicone‐based “dry‐touch” sunscreens are ideal as they reduce pore occlusion and shine while maintaining tolerability during acne therapy. A moisturizer combined with a sunscreen formulation can also benefit acne‐prone skin by improving barrier function [27, 28, 29].
2. Combination skin, with its oily T‐zone and dry cheeks, benefits from mid‐weight formulations like gel‐creams or oil‐free lotions that balance hydration and sebum control, aided by modern emulsifiers and silicones such as dimethicone [30].
3. Dry skin types require emollient‐rich cream‐based sunscreens, ideally incorporating humectants (e.g., glycerin, panthenol) to reduce transepidermal water loss and enhance barrier function, especially in xerotic or aged skin [31].
4. Sensitive skin, which is prone to stinging or allergic responses, should conduct a patch test to determine whether it is allergic to a particular sunscreen or not. If patch testing is not feasible, then use mineral sunscreens with zinc oxide or titanium dioxide due to their inert properties and low allergenicity; formulations should be fragrance‐free [32].
5. In cases of compromised or post‐procedure skin, such as post‐laser or microneedling, mineral sunscreens are preferred since they remain superficial and minimize irritation; modern micronized versions enhance cosmetic acceptability while providing broad‐spectrum protection without aggravating healing tissue [33].

### 4.3 Recommended Amount of Sunscreen

**FIGURE 4:**
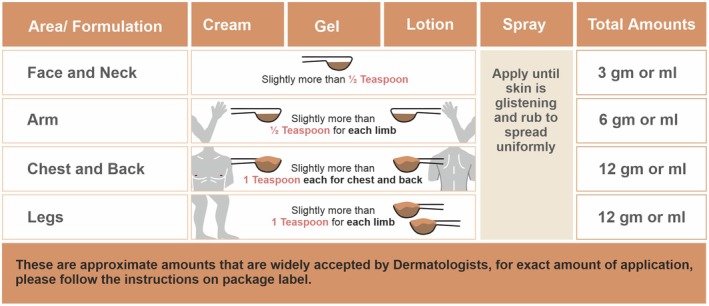
Practical recommendations for quantity of application of sunscreens [34].

To ensure optimal protection, regulatory bodies such as the FDA recommend applying 2 mg/cm^2^ of sunscreen, which equates to 35 mL per application for an average adult body (1.73 m^2^) [34, 35]. Studies show that SPF protection is directly linked to the quantity applied [35, 36, 37]. In particular, a multicentre study [36] concluded that 2 mg/cm^2^ is ideal based on the linear dependence of the SPF on the quantity applied to the skin. However, most individuals apply significantly less, typically 0.5–1.5 mg/cm^2^ which greatly reduces effectiveness [38, 39]. Figure 4 shows practical recommendations for quantity of application of sunscreens.

#### 4.3.1 Reapplication Frequency

Studies suggest that reapplying a sunscreen enhances protection, but only under certain conditions. If the recommended amount is applied initially, reapplication within 8 h is unnecessary unless the sunscreen is removed by activities such as swimming, sweating, or rubbing. While many public health agencies advise reapplying every 2–3 h, this mainly addresses under‐application and sunscreen removal due to physical activity [40, 41, 42, 43, 44].

Some studies suggest that SPF 30 sunscreens accumulate in the skin when applied multiple times daily, enhancing their protective effects [44]. Water and sweat‐resistant sunscreens have been shown to be safe and effective during exercise without interfering with sweating or body temperature regulation [45].

#### 4.3.2 Timing of Sunscreen Application

Traditional recommendations suggest applying a sunscreen 15–30 min before sun exposure, based on laboratory testing protocols [35, 40, 46]. However, a recent study confirmed that sunscreens provide protection within 10 min of application [47].

##### 4.3.2.1 Practical Recommendations

1. Sunscreens should be applied liberally to all exposed areas. As a practical estimate to the consumer, approximately 45 mL (the amount of 1 shot glass) would be more than enough to cover the entire body surface of most average‐sized individuals, or 2–3 tablespoons for the body and 1–2 teaspoons for the face and neck.
2. Reapply the sunscreen after swimming, excessive sweating, or wiping the skin. If the initial application is adequate, further reapplication may not be necessary within an 8‐h period.
3. Apply the sunscreen before sun exposure. For water‐resistant formulations, allow 15–30 min to dry before water exposure.

### 4.4 Types of Sunscreens

#### 4.4.1 Classification and Mechanism of Sunscreens

Sunscreens are categorized into organic (chemical) and inorganic (physical) filters, based on the active photoprotective ingredients in their formulation. Organic sunscreens contain carbon‐based compounds that absorb UV radiation and convert it into thermal energy, which is then released from the skin. These filters do not imply a “natural” composition, nor do they readily degrade in the environment [48].

Inorganic sunscreens, primarily composed of zinc oxide (ZnO) and titanium dioxide (TiO_2_), also absorb UV radiation but additionally scatter and reflect visible light. Their large particles create a visible white cast, forming a physical barrier on the skin. While highly effective at blocking UV radiation and visible light, traditional formulations are often considered cosmetically undesirable [48].

Recent advancements have led to the development of nanoparticle‐based inorganic sunscreens, which improve aesthetic appeal by reducing the white cast. However, micronization of these particles also diminishes their photoprotective efficacy against visible light [49]. When in nanoparticle form, inorganic sunscreens function similarly to organic filters, primarily by UV absorption.

#### 4.4.2 Sunscreen Formulations

Sunscreens are available in a variety of formulations, including creams, lotions, gels, ointments, emulsions, sprays, and sticks [50]. While labeled sun protection factor (SPF) efficacy is crucial, real‐world UV protection depends on multiple factors, such as application technique, frequency of reapplication, and user compliance. Selection of an appropriate sunscreen formulation should take into account skin feel, fragrance, cosmetic appearance, cost, and individual skin conditions [51].

Table 2 depicts different sunscreen formulations and their advantages [50, 51, 52, 53].

**TABLE 2:** Various formulations of sunscreens and their advantages.

Formulation	Advantages
Lotions and cream	Most widely used formulations Typically formulated as oil‐in‐water emulsions, which provide cost‐effective UV protection Allow easy incorporation of moisturizers, emollients, and antiaging agents Create a uniform, nontransparent sunscreen film for smooth skin application Suitable for routine daily use
Gel	Aqueous gels contain water and water‐soluble polymers, making them lightweight and easy to wash off Hydroalcoholic gels contain ethanol, which improves clarity, quick drying, and cooling sensation upon application However, ethanol‐based gels may cause skin irritation, dryness, and uneven SPF performance due to rapid evaporation Microemulsion‐based gels offer high SPF and even application, but they are expensive and may contain irritating emulsifiers
Spray	Convenient for hard‐to‐reach areas Require manual rubbing for even application, as sprays may result in uneven distribution
Sticks	Portable and lightweight, suitable for small areas and easy reapplication Formulated with wax and petrolatum, giving them a greasy texture

#### 4.4.3 Organic vs. Inorganic Sunscreen Formulations and Challenges Associated With Them

Organic sunscreens are usually formulated as lotions and creams, while other formulations include oils, gels, emulsions, mousses (fluid emulsions), aerosols, sticks, and powders. Inorganic sunscreens are more difficult to formulate due to their particulate nature. Traditionally, they were formulated as creams that were sticky, oily, and unpleasant to use, while their recent formulation as sprays received less acceptance [54].

##### 4.4.3.1 Practical Recommendations

1. The expert panel opined that the type of sunscreen formulation depends on several factors like if the sunscreen is for routine use, or for the management of a specific disorder, along with the affordability of the patient. For routine care, creams or lotions are preferred, while gel‐based formulations are preferred for acne‐prone skin type.
2. Sunscreens play a vital role in photoprotection, with various formulations tailored to different skin types, conditions, and life stages. Organic and inorganic sunscreens offer distinct mechanisms of action, with nanoparticle technology bridging the gap between efficacy and cosmetic appeal. Choice of formulation depends on aesthetic preference, application ease, and specific skin conditions.

### 4.5 Sunscreen in Dermatological Conditions

#### 4.5.1 The Role of Sunscreens in Pigmentary Disorders (Melasma & Post‐Inflammatory Hyperpigmentation—PIH)

Melasma and post‐inflammatory hyperpigmentation (PIH) are pigmentary disorders that involve an increase in melanin production in response to multiple factors [55]. Among the multiple factors implicated in its pathogenesis, sun exposure appears to play a major role in triggering and exacerbating melasma by acting directly on melanocytes [56]. Both UV radiation and visible light are considered to be involved in the pathogenesis of melasma, and hence the use of a sunscreen is strongly recommended [57]. It has been proven in multiple studies that sun protection with broad spectrum sunscreens containing UVA, UVB filters in addition to iron oxide was better and showed greater improvement in the melasma area severity index (MASI) as compared to UV filters alone [58, 59]. Thus, it is recommended to use a broad spectrum sunscreen with SPF of at least 30 that covers UVA, UVB, and VL. In order to achieve this, inorganic sunscreens are preferred as they provide protection from the entire spectrum, especially those containing iron oxide. Additionally, they also provide a camouflage effect [57]. Further, it has been shown in several studies that broad spectrum sunscreens not only improve the condition, but also enhance the depigmenting effect of other topical agents like hydroquinone [59, 60]. In addition to having good sun protection, agents having antioxidants and skin‐brightening properties like niacinamide and Sepiwhite can be considered [61, 62, 63]. The sunscreen has to be applied liberally and repeatedly throughout the day (every 2–3 h). Further, it has to be emphasized that the patient continues to use a sunscreen even when they stay indoors, as infrared light can also aggravate melasma [57].

PIH is a reactive hypermelanosis, more commonly found in skin types IV–VI [64]. Though a wide range of etiologies for PIH exists, the most common causes include acne vulgaris, atopic dermatitis, and impetigo [65]. It results from the overproduction of melanin or an irregular dispersion of pigment after cutaneous inflammation. The exact mechanism is unknown; there is a rise in melanocyte activity stimulated by cytokines, chemokines, and other inflammatory mediators as well as reactive oxygen species that are released during the inflammatory process [65]. Exposure to UV rays and VL can induce an inflammatory response stimulating melanocytes through mediators, such as reactive oxygen species, resulting in exacerbation of preexisting hyperpigmentation [55]. Thus, photoprotection is an integral part of the treatment of PIH.

A broad spectrum sunscreen with an SPF of at least 30 is recommended with sun‐protective measures, like avoidance of sun exposure and protective clothing.

#### 4.5.2 Sunscreen and Photosensitive Disorders

Photosensitive disorders are a group of conditions that manifest upon exposure to specific wavelengths of UV radiation or visible light [66]. They are divided into four major categories, which include idiopathic photo dermatoses (like Polymorphous light eruption, Solar urticaria), exogenous photochemical photo dermatoses (like Photo allergic contact dermatitis, Photo irritant contact dermatitis), genetic and metabolic photo dermatoses (like Porphyria) and photo‐aggravated photo dermatoses (like Lupus erythematosus) [67]. Regardless of the diagnosis, the management of photosensitive disorders—in most cases—will not only involve avoidance of sun exposure but also protection from sun exposure, and thus, the usage of a sunscreen becomes necessary [67]. Specific wavelengths are responsible for inciting photosensitive disorders, and hence it becomes important to identify the type of radiation and the wavelength precipitating the condition, as this information will help in choosing an appropriate sunscreen with components that absorb or reflect the specific wavelength causing the condition [68]. Broad‐Spectrum sunscreens are recommended in these conditions [69, 70, 71].

### 4.6 Sunscreens in Special Populations

#### 4.6.1 Sunscreen Use in Pediatric Populations

Early adoption of sun protection measures is essential to instill lifelong sun safety habits [72]. Infants and children under 3 years old have a thinner epidermis and stratum corneum, allowing deeper UV penetration, increasing the risk of photo damage and immune suppression [73]. UV exposure in childhood is a major risk factor for skin cancer later in life, but owing to the paucity of data on skin cancer at the population level in India, dermatologists recommend the use of a sunscreen in children on a case basis. Due to the characteristics of the skin of children, a sunscreen containing inorganic filters, like zinc oxide and titanium dioxide, is preferred over any other sunscreen [74]. A meta‐analysis of 51 studies found that childhood sunburns double the risk of developing skin cancer in adulthood [73, 75].

For infants under 6 months, direct sun exposure should be avoided, and sunscreens are not recommended.

For children over 6 months of age, sun protection strategies should include: [76]

- Avoiding direct sunlight between 10 AM and 4 PM.
- Seeking shade.
- Wearing UV‐protective clothing, hats, and sunglasses.
- Using a broad‐spectrum sunscreen (SPF 15+), applied before sun exposure and every 2 h, or more frequently if sweating or swimming is recommended.
- Inorganic (physical) sunscreens with zinc oxide and titanium dioxide are recommended, as chemical filters may contain potential allergens.

#### 4.6.2 Sunscreen Use During Pregnancy

During pregnancy, hormonal changes lead to increased skin sensitivity, pigmentation changes, and heightened risk of dermatoses such as melasma. Sun exposure should be minimized, and sunscreen use is critical [77].

However, pregnant women should be mindful of the ingredients, as certain organic UV filters may pose risks to the fetus. Certain chemical sunscreens, such as benzophenone‐3 (BP‐3 or oxybenzone), have caused concern regarding maternal‐fetal transfer [78]. BP‐3 has been detected in placental tissue, indicating that it crosses the placental barrier. A mouse study suggested that BP‐3 may alter maternal hemodynamics and impact fetal growth [79].

Sunscreens with physical UV filters are generally the safest choice [80], and consulting a dermatologist can help ensure the selection of products that are safe for use during pregnancy [78].

##### 4.6.2.1 Recommended Sunscreens for Pregnant Women

Inorganic (physical) sunscreens containing zinc oxide or titanium dioxide are preferred, as they do not penetrate the skin or enter systemic circulation. Pregnant women should consult dermatologists to select pregnancy‐safe sunscreen formulations that avoid potentially harmful organic UV filters.

###### 4.6.2.1.1 Practical Recommendations

1. Pediatric sun protection should begin in infancy, with physical sunscreens preferred for young children.
2. Pregnant women should exercise caution when selecting sunscreens, opting for physical UV filters to minimize potential fetal risks.

Future research should continue to optimize sunscreen efficacy, safety, and environmental impact, ensuring that photo protection strategies remain both effective and sustainable.

### 4.7 Potential Risks and Considerations of Sunscreen Use

#### 4.7.1 Vitamin D Deficiency

Sunscreens are formulated primarily to prevent erythema (sunburn). The action spectra for erythema and pre‐vitamin D synthesis overlap, raising concerns that sunscreen use could contribute to vitamin D deficiency [81]. Experimental studies have demonstrated that applying a sunscreen before artificial UV exposure can significantly reduce vitamin D production. However, observational studies present mixed results, with approximately 65% finding no significant association between sunscreen use and serum 25‐hydroxyvitamin D [25(OH)D] concentrations [82, 83, 84, 85, 86].

The most robust evidence comes from two randomized controlled trials (RCT) in Australia, which investigated the effect of daily sunscreen use (SPF ~16) on actinic keratosis and skin cancer prevention. These trials found no difference in serum 25(OH)D levels between individuals using a sunscreen daily and those using it intermittently or receiving a placebo [87, 88]. While no trials have specifically examined the impact of higher SPF sunscreens (SPF 50+), current data suggest that the influence of sunscreens on vitamin D levels is likely minimal.

#### 4.7.2 Nanoparticle Safety Concerns

Sunscreens containing inorganic UV filters such as zinc oxide (ZnO) and titanium dioxide (TiO_2_) often utilize these compounds in nanoparticle form. Concerns have been raised regarding their potential for systemic absorption and ROS generation, which could induce cellular damage. A recent review by the Australian Therapeutic Goods Administration (TGA, 2017) concluded that nanoparticles of ZnO and TiO_2_ do not significantly penetrate the stratum corneum, the outermost layer of the skin. Given their minimal penetration, current evidence suggests that these nanoparticles are unlikely to cause systemic harm [89].

#### 4.7.3 Hormonal Effects of Sunscreen Ingredients

Oxybenzone (benzophenone‐3), an organic UV filter commonly used in sunscreens, has raised concerns due to its potential endocrine‐disrupting properties. A systematic review of available studies found limited evidence supporting significant hormonal effects in humans [90].

Rodent studies (*n* = 7) provided inconsistent findings, with adverse effects observed only at extremely high exposure levels. Eleven human studies evaluated potential endocrine effects, with four reporting associations between urinary oxybenzone or total phenol concentrations and certain reproductive outcomes (e.g., increased male birth weight, decreased female birth weight, reduced gestational age). No significant associations were found between oxybenzone exposure and other parameters such as semen quality, fecundity, spontaneous abortion, or male genital abnormalities [90].

A RCT examining the effects of oxybenzone‐containing creams found no impact on reproductive hormone levels [91]. While further research is warranted, current data do not support strong endocrine‐disrupting effects of oxybenzone at exposure levels seen with typical sunscreens.

#### 4.7.4 Environmental Impact of Sunscreen Ingredients

Active ingredients in sunscreens, as well as their by‐products, have been detected in freshwater, coastal, and marine ecosystems, raising concerns about potential environmental toxicity. Oxybenzone and octinoxate have been shown to impair coral development, leading to coral bleaching and DNA damage [92]. These compounds can also affect the development of marine species such as sea urchins and fish, potentially disrupting aquatic ecosystems [93, 94].

The highest concentrations of sunscreen‐derived pollutants have been found in beachfront waters, particularly in areas with high levels of recreational swimming [95]. In response to these concerns, Hawaii enacted legislation banning the sale of sunscreens containing oxybenzone and octinoxate. Similar regulatory efforts have been proposed within the European Union, aiming to limit the environmental impact of chemical UV filters. New eco‐friendly sunscreen formulations are currently under development to mitigate these risks.

#### 4.7.5 The Role of Antioxidants in Sunscreen Formulations

Antioxidants are increasingly incorporated into sunscreens due to their ability to neutralize ROS, reduce cytokine‐mediated inflammation, and inhibit matrix metalloproteinase‐1 (MMP‐1) expression, which contributes to photoaging [24].

While antioxidants do not enhance SPF values or delay the onset of erythema, they provide complementary photo protection by working at deeper skin layers, unlike UV filters that primarily act at the skin's surface. Unlike sunscreens, topical antioxidants do not require continuous presence on the skin surface to exert their protective effects, making them a valuable adjunct to sun protection strategies [2, 3, 24].

##### 4.7.5.1 Section Summary

While sunscreens remain a critical tool for preventing skin cancer and photodamage, it is essential to consider potential biological and environmental effects.

Current evidence suggests that:

1. Sunscreens have minimal impact on vitamin D levels, particularly when used in accordance with public health guidelines.
2. Nanoparticles in sunscreens do not significantly penetrate the skin, reducing concerns about systemic toxicity.
3. Oxybenzone has been shown to have endocrine disruption potential in in vitro and in vivo rodent studies, with limited human data.
4. The environmental impact of chemical UV filters is a growing concern, prompting regulatory restrictions and the development of eco‐friendly alternatives.
5. Antioxidants offer additional skin protection by mitigating oxidative stress but should be used as adjuncts rather than replacements for traditional sunscreens.

In India, sunscreens are classified as cosmetics under the Drugs and Cosmetics Act of 1940, and are regulated by both the Central Drug Standard Control Organization (CDSCO) and Bureau of Indian Standards (BIS) for cosmetic product registration and licensing, and safety and performance standards, respectively. However, the BIS has not prescribed any permissible limits for UV filters as of now [96]. There is also a need for a national photoprotection policy to bridge science and practice. Additionally, future research should focus on long‐term health effects, alternative sunscreen formulations, and sustainable sun protection strategies to ensure both human and environmental safety.

## 5 Conclusion

In the evolving landscape of dermatological care, sunscreens remain one of the most accessible and impactful tools for both preventive and therapeutic skin health. This consensus underscores that sunscreen selection must go beyond SPF values—factoring in skin type, environmental context, patient age, and coexisting dermatoses to ensure both efficacy and adherence. From acne‐prone adolescents to pregnant women, and from patients with melasma to those recovering from procedures, a tailored approach ensures not just protection but comfort, cosmetic acceptability, and long‐term compliance.

Physical sunscreens continue to hold their ground in sensitive and compromised skin, while newer formulations with added antioxidants and skin‐barrier supporting ingredients are bridging protection with repair. At the same time, we must remain cognizant of ecological considerations and ingredient safety, especially as our understanding of long‐term systemic and environmental effects matures. Ultimately, sunscreen use should be positioned not merely as a seasonal precaution but as a year‐round, evidence‐backed extension of daily skin health—woven into the routine lives of individuals and guided by dermatologists with sensitivity, precision, and scientific rigor.

## Author Contributions

M.K., C.R.S., and A.S. contributed to the conceptualization and design of the study. M.K., C.R.S., A.S., A.G., A.K., G.P., I.P., D.S.K., M.S., M.V., R.K., R.D., R.R., and S.A. contributed to the acquisition of data. M.K., C.R.S., A.S., D.S.K., M.V., R.K., R.R., and S.A. contributed to the analysis of data. A.G., A.K., G.P., I.P., M.S., R.D., D.D., A.B., S.P., and H.B. contributed to the interpretation of data. M.K., C.R.S., A.S., D.S.K., M.V., R.K., R.R., and S.A. contributed to drafting the work. All authors have contributed to critically evaluating the data for intellectual content, have reviewed and approved the final manuscript, and are fully accountable for the work.

## Ethics Statement

As per Indian Council for Medical Research (ICMR) any research on “educational practices such as instructional strategies or effectiveness of or the comparison among instructional techniques, curricula, or classroom management methods,” can be exempted from ethics committee review. As per this statement there is no need for ethics committee approval in case of our manuscript. ICMR also states that “voluntary informed consent can be waived if it is justified that the research involves not more than minimal risk or when the participant and the researcher do not come into contact or when it is necessitated in emergency situations.” As per this statement there is no need for informed consent in case of our manuscript. The work was conducted according to the Declaration of the Helsinki Principles.

## Conflicts of Interest

D.D., A.B., S.P., and H.B. are employees of Glenmark Pharmaceuticals Limited. The rest of the authors have no conflicts of interest to declare.

## Supporting information


**Appendix S1:** jocd70441‐sup‐0001‐AppendixS1.xlsx.

## Data Availability

The data that support the findings of this study are available on request from the corresponding author. The data are not publicly available due to privacy or ethical restrictions.
